# Supplementary material for: Identification of a Novel Idiopathic Epilepsy Risk Locus and a Variant in the CCDC85A Gene in the Dutch Partridge Dog
Source: Animals (Basel). 2023 Feb 23;13(5):810. doi: 10.3390/ani13050810 (PMC10000155; doi:10.3390/ani13050810)
Supplement: Supplementary file 1 [file animals-13-00810-s001.zip › animals-2198567-supplementary.pdf]

animals

MDPI

Supplementary Material

File S1. Questionnaire. The translated questionnaire (the original was in Dutch) that was sent to the owners of dogs with potential idiopathic epilepsy.

A) General questions

1) Pedigree name of your dog? ______________________________________
   - Nickname (if applicable)? ______________________________________

2) When did the first seizure take place?
   - Date: ______________________________
   - Age of the dog: __________________________

3) What was the last time your dog had a seizure (date)? ________________________

4) What is the average frequency of seizures?

   a. Before starting any medication (!<u>leave empty</u> when not on medication!)

   ____________ times a day
   ____________ times per week
   ____________ times per month
   ____________ times a year

   b. The last 3-6 months

   ____________ times a day
   ____________ times per week
   ____________ times per month
   ____________ times a year

5) Compared to the beginning, the <u>frequency</u> of the seizures has been:
   - o Much reduced
   - o Reduced a little
   - o Remained the same
   - o A little increased
   - o Much increased

6) Compared to the beginning, the <u>duration</u> of the seizures has been:
   - o Much reduced
   - o Reduced a little
   - o Remained the same
   - o A little increased
   - o Much increased

7) Compared to the beginning, the <u>severity</u> of the seizures has been:
   - o Much reduced
   - o Reduced a little
   - o Remained the same
   - o A little increased
   - o Much increased

8) When does your dog have seizures the most?
   - o Morning
   - o Daytime
   - o Evening
   - o Night
   - o Variable

9) Are there factors that could trigger a seizure in your dog?
   - o Phyiscal exercise
   - o Excitation
   - o Stress
   - o Shortly after a meal
   - o After exposure to: light flashes
   - o TV screen
   - o sunlight
   - o After exposure to noise
   - o Other: ____
   - o No

10) In case of an intact bitch: does the frequency increase during heat?
   - o Yes
   - o No
   - o Not applicable

11) Is your dog already being treated for the seizures (with what and what dosage)?

12) Has a neurological examination already been performed on your dog?
   - o Yes
     Was there anything abnormal about this and what? ____________________
     ____
     ____
     Was this performed in the period surrounding a seizure (yes/no)?____________
   - o No

13) Was there ever a blood analysis done by the veterinarian?
   - o Yes
     Were there any abnormalities on this and which ones? __________________
     ____
     ____
   - o No

14) Was brain imaging done (a radiograph, CT-, or MRI scan)?
   - o Yes

Were there any abnormalities on this and which ones? ________________________
____
____

- o No

15) Was cerebrospinal fluid ever examined from your dog?
    - o Yes

      Were there any abnormalities on this and which ones? ________________________
      ____
      ____
    - o No

16) Is your dog clinically healthy between seizures?*
    - o Yes
    - o No

      Describe abnormalities: ____
      ____
      ____

** This does not include the period around a seizure (a few minutes to days before or after a seizure).*

17) Is there a known history of trauma (e.g. collision, high fall)?
    - o Yes

      Describe the trauma
    - o No

B) Description of the seizures

<u>BEFORE</u> the seizure (this may be a few minutes or even a few days before the epileptic seizure)

1) Can you predict your dog's seizures?
    - o Yes

      How long in advance
        - o __________ minutes
        - o __________ hours
        - o __________ days
    - o No (go to the question about the actual seizure)
    - o I don't know

2) Which changes do you see?
    - o Restless
    - o Barking
    - o Searching behavior
    - o Salivating
    - o Vomiting
    - o Fear

- o Wobbly gait
- o Groggy/tired
- o Aggression
- o Affectionate (more than usual)
- o Staring
- o Isolation
- o Disorientation
- o Other: ____

<u>The actual SEIZURE</u>

1) Do the seizures always look similar?
    - o Yes
    - o I don’t know
    - o No (answer the next questions for the type of seizure you see most often)

2) Try to describe the seizure in as much detail as possible:

3) What is the first thing you see at the onset of the seizure?

    ____

    If there are muscle twitches, at the level of which body part do you see this first?
    - o Head
    - o Eyes
    - o Mule
    - o Left front leg
    - o Right front leg
    - o Left hind leg
    - o Right hind leg

4) During the seizure, there are:
    - o Stiff muscles (legs)
    - o Chewing movements
    - o Clenched jaws
    - o Mouth wide open
    - o Wide pupils
    - o Convulsions/muscle tremors
        - o At the head
        - o On the front legs
        - o Whole body
        - o Other: ____
    - o Cycling movements with the leg(s)
    - o Salivating
    - o Urinating
    - o Defecation
    - o Vomiting

- o Barking
- o Other: ____
  ____

5) Does your dog respond when its name is called during a seizure?
   - o Yes
   - o No
   - o I don't know

6) Do you think your dog hears during a seizure?
   - o Yes
   - o No
   - o I don't know

7) Do you think your dog sees during a seizure?
   - o Yes
   - o No
   - o I don't know

8) How long does the seizure last?
   - o 15-30 sec
   - o 30-60 sec
   - o 1-2 min
   - o 2-5 min
   - o 5-15 min
   - o > 15 min: specify ____

<u>After</u> the seizure (a few seconds to a few days after the seizure)

1) Is your dog immediately back to normal?
   - o Yes
   - o No
   - o I don't know

2) Which abnormalities do you see after the seizure?
   - o Fear
   - o Wobbly gait
   - o Groggy/tired
   - o Aggression
   - o Affectionate
   - o Staring
   - o Blind (bumps into objects)
   - o Unease
   - o Disorientation
   - o Isolation

- Altered appetite
- Drinking more
- Other: ____

3) How long will it take for your dog to return to normal?

____

C) Additional comments

Table S1. Odds ratio calculations. Calculations comparing the number of alleles are shown in the "Alleles" table and calculations comparing genotypes in "Genotypes" table.

**Alleles**	A1	A2	OR
Case	a	b	$\frac{a * d}{b * c}$
Control	c	d	

**Geno-types**	Vt/Vt	Wt/Wt + Wt/Vt	OR
Case	A	B	$\frac{A * D}{B * C}$
Control	C	D	

A1: minor allele; A2: major allele; OR: odds ratio calculation; a: number of minor alleles in the case group; b: number of major alleles in the case group; c: number of minor alleles in the control group; d: number of major alleles in the control group; A: number of Vt/Vt cases; B: number of Wt/Wt and Wt/Vt cases; C: number of Vt/Vt controls; D: number of Wt/Wt and Wt/Vt controls.

Table S2. Overview of the *GRIK2* exons. X1 represents transcript variant X1 (XM_038684247.1) and X2 transcript variant X2 (XM_038684248.1). Exon numbers and the genomic positions of the mRNA and coding sequence (CDS) according to ROS_Cfam_1.0 (NC_051816.1) are displayed.

Exon nr	X1 mRNA		X1 CDS		X2 mRNA		X2 CDS	
	Start	Stop	Start	Stop	Start	Stop	Start	Stop
1	59766364	59766798						
2	59768851	59769006						
3	59823593	59823651						
4	59910926	59911011						
5	59980826	59980930						
6	60001027	60001080						
7	60158687	60158765						
8	60159661	60159746						
9	60162541	60162678			60183527	60183609		
10	60188919	60189325	60189211	60189325	60188919	60189325	60189211	60189325
11	60424100	60424267	60424100	60424267	60424100	60424267	60424100	60424267
12	60428314	60428571	60428314	60428571	60428314	60428571	60428314	60428571
13	60469782	60469963	60469782	60469963	60469782	60469963	60469782	60469963
14	60476652	60476705	60476652	60476705	60476652	60476705	60476652	60476705
15	60480388	60480561	60480388	60480561	60480388	60480561	60480388	60480561
16	60577431	60577574	60577431	60577574	60577431	60577574	60577431	60577574
17	60580246	60580353	60580246	60580353	60580246	60580353	60580246	60580353
18	60594627	60594740	60594627	60594740	60594627	60594740	60594627	60594740
19	60629317	60629523	60629317	60629523	60629317	60629523	60629317	60629523
20	60671103	60671326	60671103	60671326	60671103	60671326	60671103	60671326
21	60705156	60705274	60705156	60705274	60705156	60705274	60705156	60705274
22	60709593	60709810	60709593	60709810	60709593	60709810	60709593	60709810
23	60791921	60792146	60791921	60792146	60791921	60792146	60791921	60792146
24	60812912	60813162	60812912	60813162	60812912	60813162	60812912	60813162
25	60825605	60827294	60825605	60825769	60825605	60825769	60825605	60825769

Table S3. *GRIK2* primer information. Primer names and sequences, amplicon lengths, and the exons they flank are displayed. Exons are counted as displayed in *GRIK2* exons table (Table S1).

Primer name	Primer sequence	Amplicon length	Exon nr
GRIK2-F1<br>GRIK2-R1	5'-AGTGGAAGGTTGCTCCTTGGCG-3'<br>5'-AACCCGCCTGTTTACATCCAGCC-3'	368 bp	10
GRIK2-F2<br>GRIK2-R2	5'-TGCATGCATACATTAAGGTTGAGAAAA-3'<br>5'-AGGAAGTCACTAGGAACTATGGGA-3'	689 bp	11
GRIK2-F3<br>GRIK2-R3	5'-GTCTGGAAGCATTTCCCTCCTAA-3'<br>5'-TTCACACTATCTGCACACTTCATCA-3'	588 bp	12
GRIK2-F4<br>GRIK2-R4	5'-GTGACAACCCTTTGGGGTACA-3'<br>5'-CCCCCTCCAACTCTCCCC-3'	627 bp	13
GRIK2-F5<br>GRIK2-R5	5'-AGATGTGCTAATTTCAGGTTTGCCTCA-3'<br>5'-CCAGTCATTCTCACTCAAAAGCCCA-3'	453 bp	14
GRIK2-F6<br>GRIK2-R6	5'-AGCCTGCTGTTCCATTGGTGG-3'<br>5'-GCAGTCCTGATACCATGCCTTTAATGT-3'	540 bp	15
GRIK2-F7<br>GRIK2-R7	5'-TGCACTTATCCTGTGTGTGTGTGC-3'<br>5'-AGGAAGGTGAAAACTAACAATGCCGT-3'	803 bp	16
GRIK2-F8<br>GRIK2-R8	5'-CTGTAGACAAAAATCCTCCAAAGCTCA-3'<br>5'-ATGACCTGCTCCAAATAGGAAAGTCTA-3'	634 bp	17
GRIK2-F9<br>GRIK2-R9	5'-CGACAGCAGATAATGCAGCAGAGGT-3'<br>5'-AGTAAAGTTCACATGCAGTCCCTCACA-3'	595 bp	18
GRIK2-F10<br>GRIK2-R10	5'-TCTGCCTTCTTAGGATTGCAAACTG-3'<br>5'-AGGCATCACTCATTCTTTCTGCTG-3'	727 bp	19
GRIK2-F11<br>GRIK2-R11	5'-AGCAGGTCCTCCAATTCAGGATCAA-3'<br>5'-ACTGTTTTGCTTCCTGGCTCAAGAGA-3'	715 bp	20
GRIK2-F12<br>GRIK2-R12	5'-CAGTTGCTGTTGTGCATGTGAAATTGT-3'<br>5'-TGCATTAGGTAGATGGAGAAGCTGGA-3'	420 bp	21
GRIK2-F13<br>GRIK2-R13	5'-TGCTCTCCCAATGGTTGTGGC-3'<br>5'-ACGAGAGAAAGGTTTGTTTGCACTCT-3'	448 bp	22
GRIK2-F14<br>GRIK2-R14	5'-GGAACTGCTACAAGGAACTCTTTTGC-3'<br>5'-CTCACCTATTTAGAAGCAGATGTCCCA-3'	765 bp	23
GRIK2-F15<br>GRIK2-R15	5'-ACATTGGCATACCCTGGTGCATCT-3'<br>5'-TGGCGGAAGACAATTAGCGGTCA-3'	483 bp	24
GRIK2-F16<br>GRIK2-R16	5'-TGTATCTGTGCATTCCTTGTTTGCAGT-3'<br>5'-GGGGCCAGTAACATCACCACCT-3'	905 bp	25

Table S4. PCR and sequencing information. PCR/sequencing mixes and programs used for *GRIK2* CDS sequencing

**PCR Mix:**<br>5.7 µl H_2O<br>1.0 µl 10x Key buffer<br>1.0 µl Primers (5 µM each)<br>0.2 µl dNTPs (10 mM each)<br>0.1 µl TEMPase Hotstart DNA polymerase (5 U/µl)<br>2.0 µl Template<br>10.0 µl Total volume	**PCR Program:**<br>14'30'' - 95°C<br>00'30'' - 95°C]<br>00'30'' - 62°C] x 35<br>01'00'' - 72°C]<br>04'00'' - 72°C<br>Hold - 15°C
**Sequencing mix:**<br>3.0 µl H_2O<br>2.0 µl 5x SEQ-buffer<br>1.5 µl Sequencing primer (2 µM)<br>1.0 µl GC-rich<br>0.5 µl RR-mix<br>2.0 µl Template<br>10.0 µl Total volume	**Sequencing Program:**<br>2'00'' - 95°C<br>0'20'' - 95°C]<br>0'10'' - 60°C] 30x<br>4'00'' - 65°C]<br>Hold - 15°C

Table S5. Candidate variants genotyping - primer information. Primer names and sequences, amplicon lengths, and the primer used for sequencing are displayed.

Primer name	Primer sequence	Amplicon length	Sequencing primer
ENAH-F1	5'-CTTTTTAATGTTTGGTTTTCAGGCA -3'	Wt: 200 bp<br>Vt: 185 bp	N.A.*
ENAH-R1	5'-CTCCCGCTCTCGGTCCAG-3'		
CCDC85A-F1	5'-GAGACTGGGCCGCTACACGG-3'	398 bp	CCDC85A-F1
CCDC85A-R1	5'-GGAGATGCTCGGGGCTGGTG-3'		
VPS54-F1	5'-GCTTGAGGGATTTTGGATAAGGGGTCT-3'	276 bp	VPS54-R1
VPS54-R1	5'-ACCTGTGGGTTCCTTGGGACAT-3'		
SPAST-F1	5'-TAAAGCAGGACAAAAAGAGCAAGC-3'	354 bp	SPAST-F1
SPAST-R1	5'-AAAGCGGGTCAGAGAAGACAC-3'		
BRINP3-F1	5'-GTCACTCTGGAGACGCTACATCAAC-3'	321 bp	BRINP3-R1
BRINP3-R1	5'- AGGGTATGAGATGCCTTGACCCA-3'		

*Genotyping was based on amplicon length viewed on gel electrophoresis instead of sequencing.

Table S6. Associated SNPs on chromosome 12 based on the univariate linear mixed model. (SNP) SNP name, (BP) base pair position on chromosome 12 in ROS_Cfam_1.0, (A1) minor allele, (F_A) frequency of A1 in IE affected dogs, (F_U) frequency of A1 in unaffected dogs, (A2) major allele, (P_{raw}) raw p-values, (P_{adj}) Bonferroni-adjusted p-values, (OR) odds ratio, (CI_L) lower bound of the 95% confidence interval (CI_U), upper bound of the 95% CI.

SNP	BP	A1	F_A	F_U	A2	P_{raw}	P_{adj}*	OR	CI_L	CI_U
BICF2G630119560	60299232	A	0.6875	0.1744	G	4.40E-07	0.044	10.4	4.1	26.5
BICF2P1418795	60231951	C	0.5	0.1047	A	9.80E-06	0.033	8.6	3.2	22.8
TIGRP2P170058_rs8611580	59758472	A	0.5	0.1047	G	9.80E-06	0.033	8.6	3.2	22.8

* Values above the dotted line are calculated on genome-wide level and those below on chromosome-wide level.

Table S7. Preliminary genotyping results for the candidate variants. The number of Wt/Wt, Wt/Vt, and Vt/Vt dogs, as well as the variant allele frequency (Vt%) are shown for all cases and selected controls.

	**Cases**				**Controls**			
	Wt/Wt	Wt/Vt	Vt/Vt	Vt%	Wt/Wt	Wt/Vt	Vt/Vt	Vt%
ENAH	13	4	1	16.7%	11	7	0	19.4%
CCDC85A*	11	2	5	33.3%	16	2	0	5.6%
VPS54	9	8	1	27.8%	13	5	0	13.9%
SPAST*	11	6	1	22.2%	17	1	0	2.8%
BRINP3	16	0	2	11.1%	14	3	1	13.9%

* Significant odds ratio > 5.

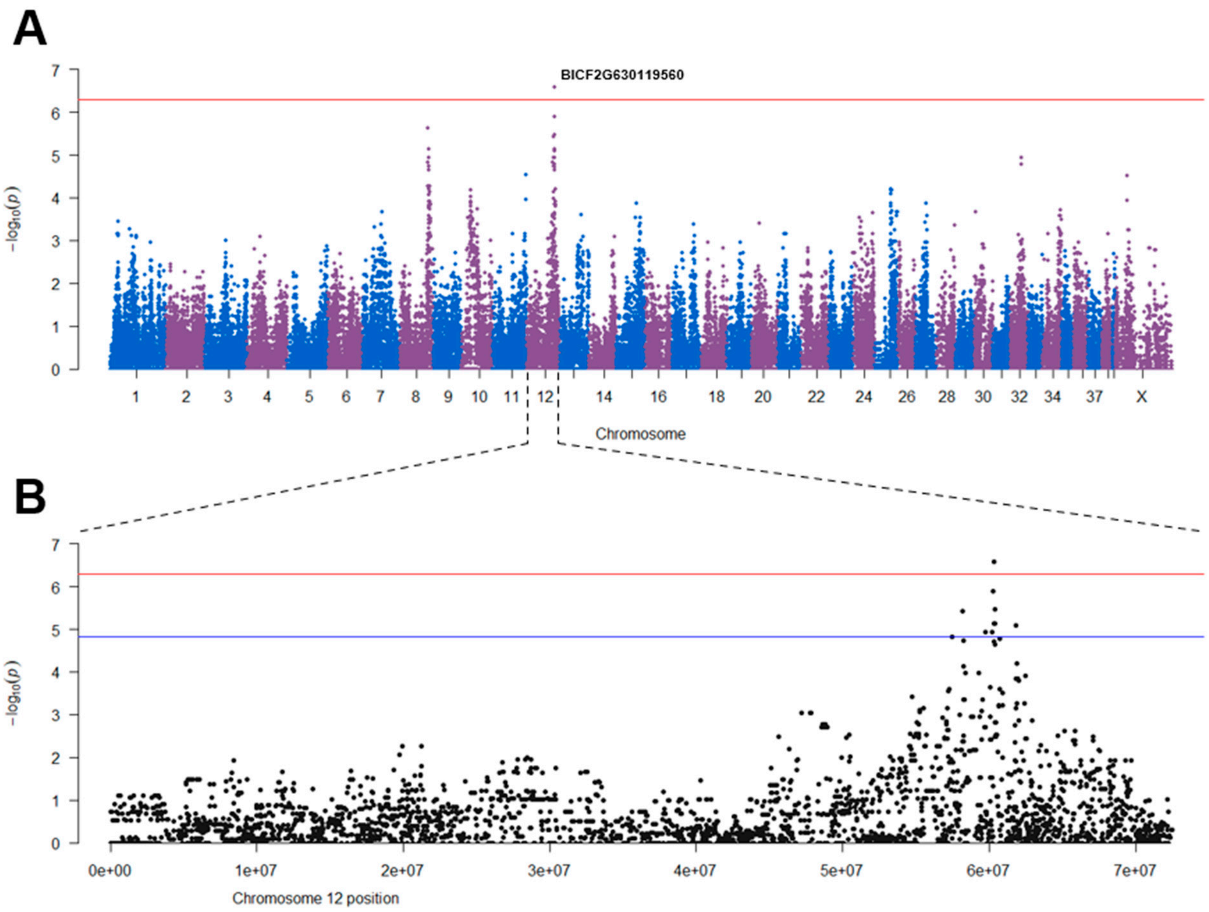

Figure S1. Manhattan plots after Fisher exact testing. A) Manhattan plot with the red line indicating the genome-wide significance threshold. B) Manhattan plot zoomed in on chromosome 12. The red line indicates the genome-wide significance threshold and the blue line indicates the chromosome-wide significance threshold.

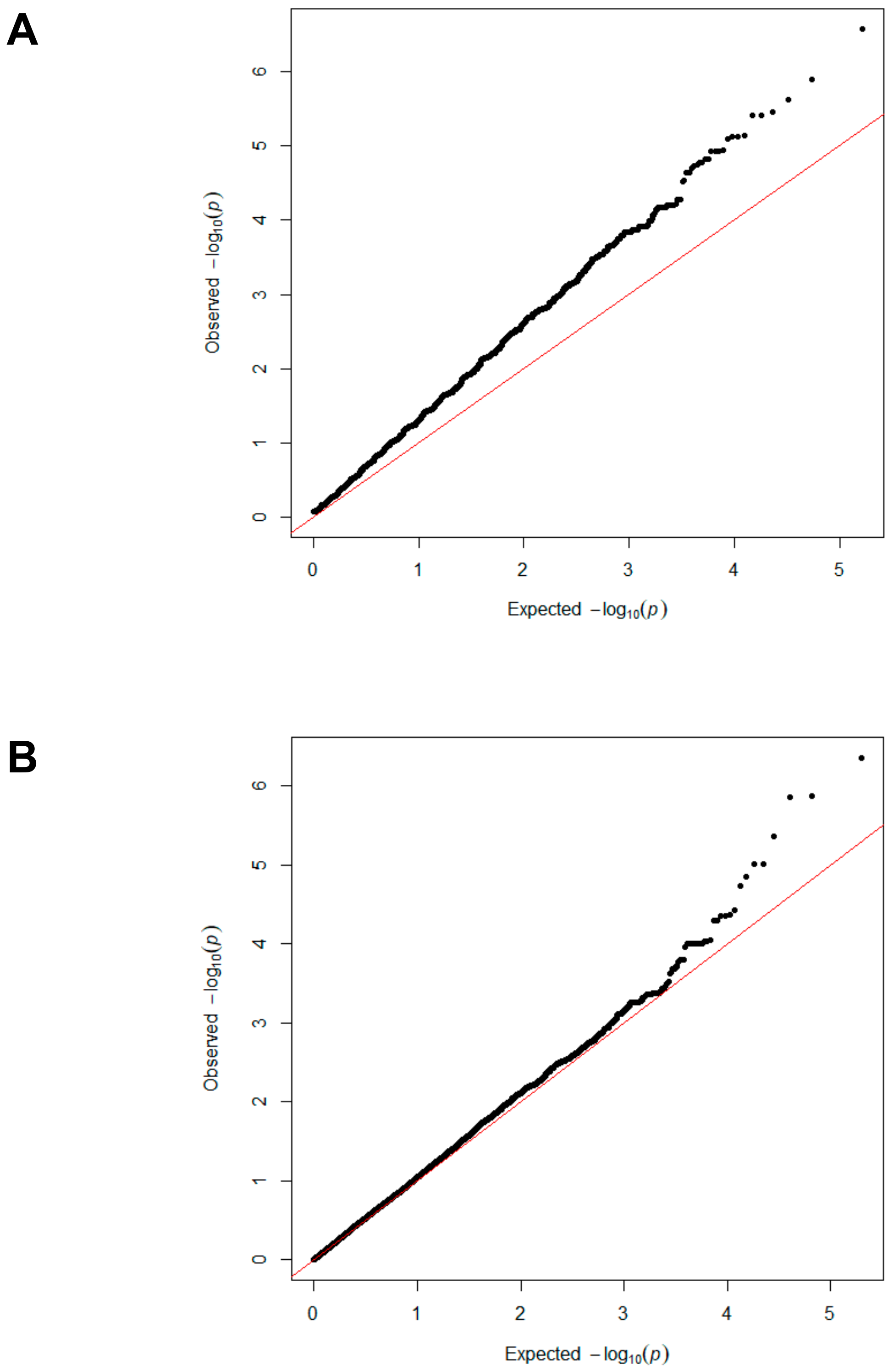

Figure S2. QQ plots of the expected and observed -log(p) values. A) QQ plot generated with the observed -log(p) values following Fisher exact testing. B) QQ plot generated with the observed -log(p) values following Univariate Linear Mixed Model testing.

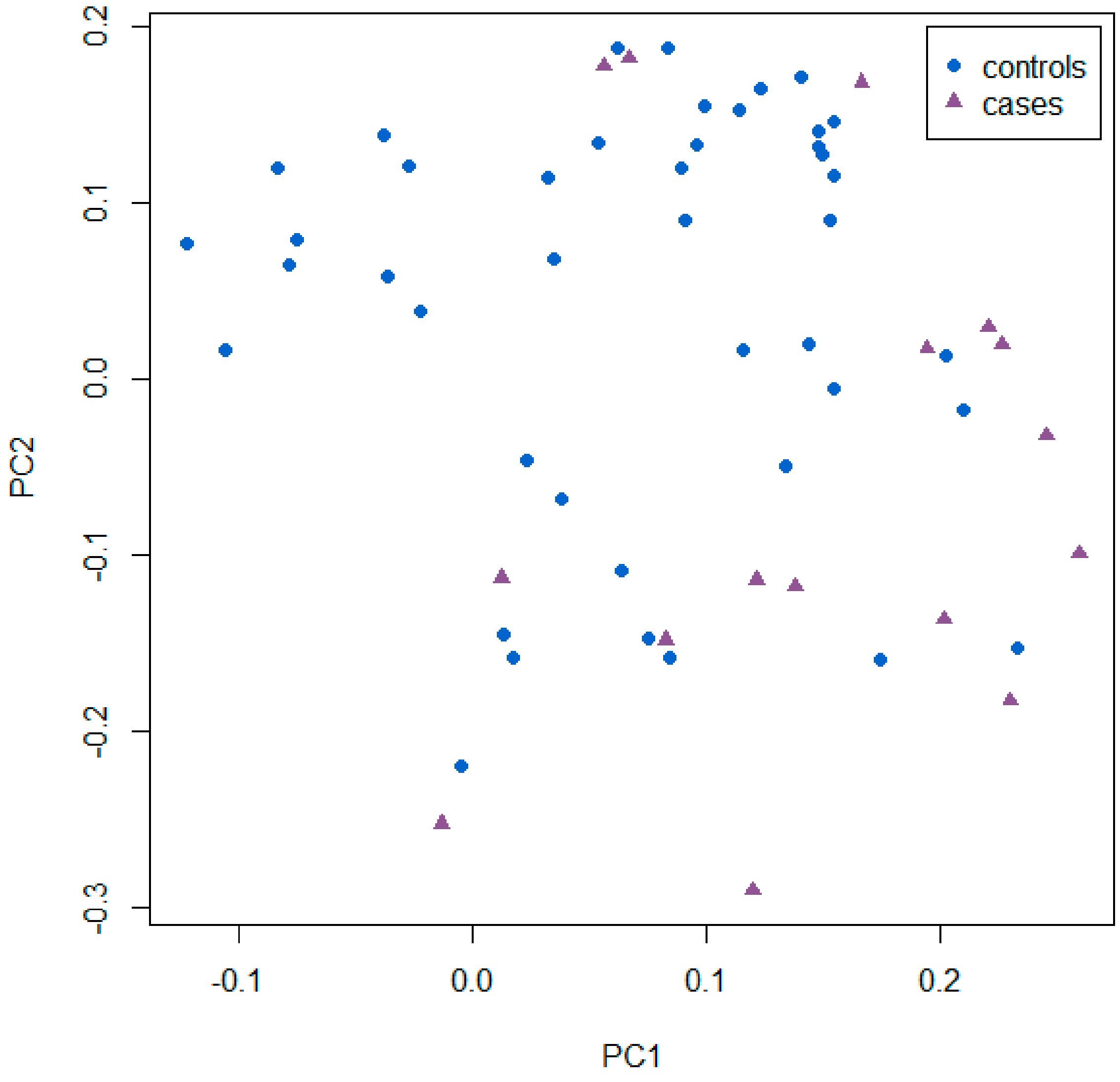

Figure S3. PCA plot of the 57 dogs retained in the GWAS analysis. (PC1) principal component 1, (PC2) principal component 2.
